# ID 55 - Efeito do Uso dos Dados de Registros de Lesões na Elaboração de Plíticas, nas Hospitalizações e na Mortalidade: uma revisão sistemática

**DOI:** 10.5327/2237-9622.2025.v34s1.55

**Published:** 2025-11-25

**Authors:** Ana Cláudia Medeiros de Souza, Luana Emanuelly Sinhori Lopes, Tayna Felicíssimo Gomes de Souza Bandeira, Lucas Reis Correia, Naiza Nayla Bandeira de Sá, Bruno Zoca de Oliveira

**Keywords:** sistema de registros, formulação de políticas, política de saúde, ferimentos e lesões, mortalidade, mortalidade hospitalar, hospitalização

## Abstract

**Introdução:**

Iniciado em 2021, um projeto brasileiro tem como objetivo estabelecer um registro nacional de lesões que compila dados sobre eventos e indivíduos de todo o País, independentemente da gravidade das lesões. O registro integra informações do atendimento pré-hospitalar e hospitalar, de diversos sistemas de saúde que carecem de interoperabilidade e dados de setores como bombeiros e polícia. Seu objetivo principal é aprimorar a vigilância em saúde, fornecendo informações oportunas e de alta qualidade que orientem estratégias de prevenção e informem a formulação de políticas. Além disso, o projeto busca reduzir a morbidade e mortalidade associadas a lesões. Considerando os esforços atuais para implementar um registro de lesões no Brasil, este estudo investiga os efeitos dos dados do registro de lesões na formulação de políticas, taxas ou duração de hospitalização e mortalidade.

**Método:**

A revisão sistemática seguiu as diretrizes PRISMA, com um protocolo registrado no PROSPERO. Cinco bases de dados foram pesquisadas em novembro de 2023, com uma atualização realizada em março de 2024, incorporando listas de referências dos estudos incluídos. Dois revisores analisaram independentemente os registros, extraíram dados e avaliaram a qualidade metodológica usando a ferramenta Newcastle-Ottawa Scale (NOS), resolvendo desacordos com um terceiro revisor. Os estudos eram elegíveis se relatassem resultados relacionados à implementação e uso de dados de registro de lesões ou traumas para pelo menos um desfecho de interesse, enquanto aqueles baseados em outras fontes foram excluídos. A síntese dos resultados foi apresentada em tabelas, e os efeitos observados foram relatados como diferenças em número ou porcentagem.

**Resultados:**

De 9.100 estudos recuperados, 3.951 foram excluídos devido à duplicação, restando 5.149 para seleção, com 15 textos completos revisados. Apenas cinco estudos atenderam aos critérios de inclusão, destacando uma escassez notável de pesquisas sobre os efeitos dos dados do registro nos desfechos de lesões. É importante notar que os estudos incluídos refletem correlações em vez de causalidades, e atualmente não há publicações sobre impacto. Os achados sugerem que os registros de lesões e traumas influenciam positivamente a formulação de políticas, o que, por sua vez, melhora os desfechos de saúde. Um estudo observou redução de três dias na permanência na unidade de terapia intensiva e uma redução de 4,1% na mortalidade hospitalar esperada (de 22,8% para 18,7%) para pacientes com um Escore de Gravidade de Lesão (ISS) ≥16, enquanto outro mostrou diminuição anual de 42% nas internações hospitalares por lesões de trânsito (de 45 para 16). A heterogeneidade metodológica significativa e o pequeno número de estudos limitaram a viabilidade de uma metanálise.

**Conclusão:**

Até onde sabemos, esta é a primeira revisão sistemática que visa mapear os efeitos relacionados aos dados do registro. Pesquisas anteriores concentraram-se predominantemente nessa relação com sistemas de trauma ou para gerar conhecimento científico, em vez de avaliar o impacto ou os efeitos do registro em si. Em conclusão, o estabelecimento de um registro de lesões no Brasil apresenta uma oportunidade significativa para melhorar os resultados de saúde por meio da formulação de políticas informadas. Embora os efeitos diretos sobre a morbidade e a mortalidade possam não ser imediatamente evidentes, o papel do registro em facilitar medidas preventivas e melhorar as capacidades de vigilância é inestimável.

